# Impact of prebiotic supplementation on the zootechnical and health performance in broiler chickens

**DOI:** 10.17221/37/2025-VETMED

**Published:** 2025-12-19

**Authors:** Maroua Mansouri, Nabila Hammami, Achour Yahia, Khelaf Saidani, Zoubida Boumahdi, Dalila Tarzaali, Nora Mimoune

**Affiliations:** ^1^Institute of Veterinary Sciences, SAAD Dahlab University Blida 1, Blida, Algeria; ^2^Laboratory of Protection and Valorisation of Agrobiological Resources, Department of Biotechnology and AgroecologyFaculty of Natural Sciences and Life, SAAD Dahlab University Blida 1, Blida, Algeria; ^3^Biotechnologies Laboratory Related to Animal Reproduction (LBRA), Institute of Veterinary Sciences, SAAD Dahlab University Blida 1, Blida, Algeria; ^4^Biotechnologies Platform for Animal Medicine & Reproduction, SAAD Dahlab University Blida 1, Blida, Algeria; ^5^Animal Health & Production (SPA) Laboratory, Higher National Veterinary School (ENSV), Algiers, Algeria

**Keywords:** chicken, lactic acid bacteria, mannan-oligosaccharide, prebiotic, zootechnical performance

## Abstract

This study aimed to evaluate the effectiveness of mannan-oligosaccharides (MOSs) in broiler chicken feed throughout the complete rearing cycle, focusing on zootechnical performance and lactic acid bacteria. Over a period of 49 days, a total of one thousand and eighty (1 080) day-old ISA 15 chicks were divided into two (control and experimental) groups of 540 subjects each (9 replicates of 60 chicks per treatment). They were fed the same basic diet, but only the experimental group received a yeast cell wall extract-based prebiotic (AGRIMOS^®^, France), administered continuously at a dose of 2 g/kg throughout the different rearing phases. Under our local conditions, the prebiotic supplementation resulted in a significant increase in body weight gain (*P* < 0.01), reaching 1 559.82 ± 41.47 g during the growth phase and 913.20 ± 72.58 g during the finishing phase. Moreover, a significant reduction in the feed conversion ratio was observed throughout the rearing cycle (*P* < 0.05). Across all segments of the analysed intestinal tract (duodenum, ileum, and caecum), the data showed that chickens supplemented with the prebiotic had a significantly higher number of lactic acid bacteria than the control group at the start, growth, and finishing phases (*P* < 0.01). Our findings demonstrated a clear impact of the prebiotic on the feed utilisation under our rearing conditions, which required further studies to elucidate the underlying mechanisms of action.

The poultry industry primarily aims to improve the zootechnical performance of chickens to produce high-quality final products (Yadav and Jha 2019). In recent decades, changes in poultry production management systems have led to the reduction or even the ban of antibiotic use as growth promoters in animal feed (Park et al. 2019; Jeni et al. 2021) in response to a growing consumer demand for antibiotic-free poultry products (Ahiwe et al. 2021). The concept of “gut health” is currently generating increased interest in veterinary literature, particularly in the poultry sector (Cummings et al. 2004). The condition and proper functioning of the gastrointestinal tract play a key role in the health and performance of broiler chickens (Clavijo and Florez 2018). Modulating the gut microbiota has become one of the most promising strategies for improving the performance in poultry production (Tiseo et al. 2020). To address these concerns, researchers have turned toward natural and sustainable alternatives to antibiotics as growth promoters (Abd El-Hack et al. 2022b). Solutions, such as prebiotics, probiotics (Karar et al. 2023), symbiotics (Ammari et al. 2022), enzymes, essential oils (Abd El-Hack et al. 2022a), organic acids (Mimoune et al. 2023), and plant extracts, have been developed to improve gut health and poultry performance (Chowdhury et al. 2023). Natural feed additives, such as prebiotics (Willis and Reid 2008), are considered a potential alternative to antibiotic growth promoters (AGPs) in poultry nutrition (Yang et al. 2009). Prebiotics, as defined by Gibson et al. (2004), are non-digestible food substrates that selectively stimulate the growth or metabolic activity of beneficial microorganisms in the colon, thereby improving gut health. These compounds are indigestible by the poultry gastrointestinal tract and fermentable by beneficial bacteria such as *Lactobacillus* and *Bifidobacterium* (Ricke et al. 2023). Among the most commonly studied and used prebiotics in poultry feed are inulin, fructo-oligosaccharides (FOSs), and mannan-oligosaccharides (MOSs). Refined functional carbohydrates (RFCs), including MOSs, β-glucans, and d-mannose, constitute a category of prebiotics used in both animal and human nutrition (Dallies et al. 1998). Notably, MOSs, derived from the structural components of the yeast *Saccharomyces cerevisiae* cell walls, are essential additives known for their beneficial effects on poultry growth and physiology (Teng and Kim 2018). β-glucans have garnered considerable interest due to their biological properties, including toxin adsorption, pathogen agglutination, and immune modulation (Teng and Kim 2018; Hernandez-Ramirez et al. 2021; Papp et al. 2021). MOSs are prebiotic substances derived from yeast cell walls, specifically from the mannans present in *Saccharomyces cerevisiae*. These oligosaccharides are indigestible by poultry but are selectively fermented by the beneficial gut bacteria, thereby promoting a healthy intestinal microbiota (Chacher et al. 2017). These approaches generally enhance the beneficial bacterial flora in the poultry gut (Micciche et al. 2018) while limiting the population of intestinal pathogens (Kim et al. 2019). They also contribute to maintaining efficient poultry production (Abd El-Hack et al. 2022b), improving the gut health and broiler performance (Karar et al. 2023), and supporting the production of antibiotic-free meat (Chowdhury et al. 2023). This study was conducted in response to growing global concerns over the overuse of antibiotics in poultry farming and the resulting rise in antimicrobial resistance. While previous research has assessed the effects of prebiotics on poultry performance, this current work aimed to evaluate a specific commercial formulation (AGRIMOS^®^) under conditions that reflect the Algerian poultry sector. The purpose was to explore natural alternatives to reduce antibiotic dependence, offering a relevant and practical solution to improve poultry health and productivity in Algeria. More particularly, the objective of this study was to evaluate the impact of dietary supplementation with mannan-oligosaccharides (MOSs) and β-glucan (AGRIMOS^®^, France) on broiler chickens, explicitly focusing on the growth performance parameters including the body weight, feed intake, feed conversion ratio, and mortality as well as on the enumeration of the lactic acid bacteria in the digestive tract over a complete production cycle

## MATERIAL AND METHODS

### Animals

The study was conducted over a period of 49 days, involving a total of one thousand and eighty (1 080) day-old chicks (strain ISA 15, mixed sexes) from the same hatchery. The chicks, individually weighed on reception (mean weight: 36.5 g), were divided into two groups of 540 subjects each. Each group was further divided into nine replicates of 60 chicks. The control group (C) received a standard diet formulated to meet the nutritional requirements of each rearing phase: start (days 1–10) with 2 800 kcal/kg metabolisable energy (ME) and 21% crude protein (CP), growth (days 11–37) with 2 900 kcal/kg ME and 19% CP, and finishing phase (days 38–47) with 2 930 kcal/kg ME and 17% CP. The treated group (P) was fed the same diet continuously supplemented with a commercial yeast cell wall extract (AGRIMOS^®^, Lallemand, France), rich in mannan-oligosaccharides (MOSs) and β-glucans, at a dosage of 2 kg per tonne of feed, or 2 g/kg (Awaad et al. 2011). All the animals were reared in the same building under controlled environmental conditions (temperature, ventilation, and lighting), with *ad libitum* access to feed and water.

### Variables measured in the study

Zootechnical performance indicators, including the live body weight, average daily gain, feed intake, feed conversion ratio (FCR), and mortality rate, were recorded on days 10, 38, and 47. For the study of the intestinal lactic acid bacteria, 18 chicks per group (2 per replicate) were sacrificed on days 10, 38 and 47. The digestive tract was quickly and aseptically removed. The intestine was then divided into three sections: duodenum, ileum, and caeca. For the chicks sacrificed on days 10 and 38, 1 g of each intestinal segment was aseptically collected and placed into stomacher bags containing 9 ml of a tryptone salt extraction (TSE) broth, constituting the mother suspension. For the day 47 samples, this suspension was prepared using 0.5 g of each segment diluted in 45 ml of the TSE broth. Serial decimal dilutions were prepared in test tubes up to 10^–5^.

Then, 1 ml of the last two dilutions was deep-plated in duplicate into sterile Petri dishes containing melted and cooled MRS (de Man, Rogosa, and Sharpe) agar. The plates were incubated under anaerobic conditions at 37 °C for 72 h to allow enumeration of lactic acid bacteria colonies. Only the plates showing well-developed, clearly separated colonies free from yeast or mould contamination were selected for further evaluation of their appearance, shape, size, colour, and colony count. Typical colonies (0.5–1 mm in diameter, whitish or opaque, sometimes translucent, milky, or creamy in appearance, with smooth, circular, convex, and regularly bordered morphology) were examined by Gram staining to confirm the presence of Gram-positive lactic acid bacteria.

The enumeration of the lactic acid bacteria was performed by using the following formula:

$$\text{Number of bacteria/sample = }\frac{n_{c-1}+n_{c}}{1.1\times 10^{-(x-1)}}$$ (1)

where:

*n*_c–1_ – number of colonies obtained from a dilution of 10^–(x–1)^, here 10^–4^;

*n*_c_ – number of colonies obtained from a dilution of 10^–x^, here 10^–5^.

The results were expressed as log_10_ CFUs/g (Colony Forming Units per gram), a standard unit used to quantify the number of viable bacteria in one gram of sample on a logarithmic scale.

### Ethical statement

All animal studies were conducted with the utmost regard for animal welfare, and all animal rights were appropriately protected. No animal suffered during the course of the work. All experiments were carried out in accordance with the guidelines of the Institutional Animal Care Committee of the Algerian Higher Education and Scientific Research (Agreement No. 45/DGLPAG/DVA.SDA. 14).

### Statistical analyses

The results were expressed as the mean ± stan-dard deviation (SD). The Shapiro–Wilk test was used to assess normality, following Kappes et al. (2020), before performing parametric tests such as the Student *t*-test and a variance analysis. A one-way nonparametric analysis of variance (ANOVA) was used to compare groups (Kruskal–Wallis test) when the ANOVA assumptions were not met. Four quantitative variables (average weight gain, feed conversion rate, mortality rate, and lactic acid bacteria enumeration) and two factors and their combinations were considered: rearing phase, treatment type, and the use or absence of prebiotics. Tukey’s multiple-comparison test was applied for the post hoc comparison of the means. To perform multifactorial analyses of variance, the non-Gaussian-distributed variables were transformed using the square root function rather than the Napierian logarithm, which would yield negative values. The same statistical analyses were also applied to the cumulative results of the three rearing phases: start, growth, and finishing. Data were analysed using the open-access (R Core Team, 2023) statistical software v4.3.1.

## RESULTS

### Mortality rate

Our results indicate that prebiotic supplementation reduced mortality rates during both the growth and finishing phases compared with the control group (0.19 vs 1.13 in the growth phase, and 0.59 vs 1.36 in the finishing phase). Although these differences were not statistically significant (ns), the cumulative mortality rate was lower in the prebiotic group (1.89) compared to the control group (3.60). This suggests that prebiotics may have some protective effect on the broiler health, although the *P* = 0.187 indicates that the difference did not reach statistical significance (Table 1; Table 2).

**Table 1:** Effect of prebiotics on mortality rate (*n* = 9)

Phase	Treatment	Mortality rate (mean ± SD)	ANOVA (*P*)
Start	control	1.1 ± 1.37	ns
	prebiotics	1.1 ± 1.18	
Growth	control	1.13 ± 1.85	ns
	prebiotics	0.19 ± 0.56	
Finishing	control	1.36 ± 1.61	ns
	prebiotics	0.59 ± 0.89	

ns = not significant; SD = standard deviation

**Table 2:** Effect of prebiotics on average weight gain/feed conversion/mortality across the cumulative period (*n* = 9)

Phase	Treatment	Cumulative period	ANOVA (*P*)
Mortality rate (mean ± SD)	control	3.60 ± 3.36	*P* = 0.187
	prebiotics	1.89 ± 1.59	
Feed conversion (mean ± SD)	control	2.49 ± 0.18	**
	prebiotics	2.27 ± 0.10	
Average weight gain (mean ± SD)	control	2 311.09 ± 85.43	***
	prebiotics	2 630.93 ± 48.45	

**P < 0.01; ***P < 0.000

SD = standard deviation

### Feed conversion ratio (FCR)

The results for feed conversion ratio (FCR) showed significant improvements in the prebiotic-supplemented group across the various rearing phases. During the start phase (D1–D10), the broilers receiving prebiotics exhibited a significantly lower FCR (2.12) compared to the control group (2.19) (*P < *0.05). This improvement was even more pronounced during the growth phase (D11–D37), with an FCR of 2.23 in the supplemented group versus 2.54 in the control group (*P < *0.001), indicating enhanced feed efficiency. In the finishing phase (D38–D47), the FCR remained significantly lower in the prebiotic group (2.39) compared to the control (2.46) (*P* < 0.05). The cumulative FCR over the entire experimental period (D1–D47) showed a substantial improvement in the prebiotic group (2.27) relative to the control group (2.49), with a high level of statistical significance (*P < *0.01) (Table 3; Table 2).

**Table 3:** Effect of the prebiotics on the feed conversion rate across the rearing phases (*n* = 9)

Phase	Treatment	Feed conversion (mean ± SD)	ANOVA (*P*)
Start	control	2.19 ± 0.08	*
	prebiotics	2.12 ± 0.17	
Growth	control	2.54 ± 0.31	***
	prebiotics	2.23 ± 0.15	
Finishing	control	2.46 ± 0.14	*
	prebiotics	2.39 ± 0.14	

*P < 0.05; ***P < 0.001

SD = standard deviation

### Average body weight gain

The most pronounced improvement was observed in average body weight gain, with the prebiotic-supplemented group demonstrating significantly higher performance across all production phases. During the start phase, chicks receiving prebiotics had an average gain of 157.91 g, compared with 150.90 g in the control group (*P < *0.05). In the growth phase, the difference widened markedly, with the supplemented group reaching 1 559.82 g versus 1 384.41 g in the control group (*P < *0.001). This trend continued in the finishing phase, where the prebiotic group recorded a gain of 913.20 g, significantly exceeding the 775.78 g observed in the control group (*P < *0.01). Cumulatively, the total body weight gain was substantially higher in the supplemented group, reaching 2 630.93 g compared to 2 311.09 g in the control group (*P < *0.001), corresponding to a global enhancement in the growth performance of approximately 13.8% (Table 4; Table 2).

**Table 4:** Effect of the prebiotics on the average weight gain (*n* = 9)

Phase	Treatment	Average weight gain (mean ± SD)	ANOVA (*P*)
Start	control	150.90 ± 9.70	*
	prebiotics	157.91 ± 13.80	
Growth	control	1 384.41 ± 81.88	***
	prebiotics	1 559.82 ± 41.47	
Finishing	control	775.78 ± 54.78	**
	prebiotics	913.20 ± 72.58	

*P < 0.05; **P < 0.01; ***P < 0.001

SD = standard deviation

### Lactic acid bacteria count

Dietary supplementation with prebiotics resulted in a significant increase in lactic acid bacteria counts across all examined intestinal segments. In the duodenum, a progressive rise in the lactic acid bacteria concentration was observed throughout the rearing phases. In the control group, values ranged from 6.32 ± 0.20 to 6.49 ± 0.27 log_10_ CFU/g, whereas in the prebiotic-treated group, they increased from 6.40 ± 0.25 to 6.62 ± 0.27 log_10_ CFU/g. This difference was statistically significant (*P < *0.01), suggesting a positive impact of the prebiotics on duodenal lactic acid bacteria colonisation. A similar trend was observed in the ileum, with lactic acid bacteria counts rising from 6.49 ± 0.31 to 6.74 ± 0.09 log_10_ CFU/g in the control group and from 6.6 ± 0.19 to 6.86 ± 0.04 log_10_ CFU/g in the prebiotic group (*P < *0.01). These findings support the role of prebiotics in maintaining and enhancing the beneficial microbiota in the small intestine. In the caecum, a significant site of microbial fermentation, prebiotics also significantly elevated lactic acid bacteria populations, reaching 6.88 ± 0.05 log_10_ CFU/g at the finishing stage, compared to 6.67 ± 0.12 log_10_ CFU/g in the control group (*P < *0.01). Collectively, these results demonstrate that prebiotic supplementation promotes the favourable modulation of the intestinal microbiota by stimulating the growth of lactic acid bacteria throughout the production cycle, with the most pronounced effects observed in the later stages (Table 5).

**Table 5:** Effect of the prebiotics on the lactic acid bacteria count (*n* = 18)

Enumeration of lactic acid bacteria (log_10_ CFU/g)	Treatment	Start	Growth	Finishing	ANOVA (*P*)
In the duodenum (mean ± SD)	control	0.32 ± 0.20	6.43 ± 0.26	6.49 ± 0.27	**
	prebiotics	6.40 ± 0.25	6.50 ± 0.30	6.62 ± 0.27	
In the ileum (mean ± SD)	control	6.49 ± 0.31	6.69 ± 0.10	6.74 ± 0.09	**
	prebiotics	6.68 ± 0.19	6.74 ± 0.08	6.86 ± 0.04	
In the caeca (mean ± SD)	control	6.56 ± 0.08	6.60 ± 0.07	6.67 ± 0.12	**
	prebiotics	6.60 ± 0.11	6.65 ± 0.09	6.88 ± 0.05	

**P < 0.01

SD = standard deviation

## DISCUSSION

The current study was conducted to assess the effects of mannan-oligosaccharides (MOSs) and β-glucan (AGRIMOS^®^, France) on the zootechnical and sanitary performance of broiler chickens. Regarding mortality, although no statistically significant difference was observed, the group supplemented with MOSs showed a non-significant reduction in mortality. These findings are consistent with previous studies (Kim et al. 2011; Pourabedin et al. 2014; Ding et al. 2019; Kamran et al. 2021), which similarly reported no significant effect of MOSs on mortality. Conversely, other research, such as that conducted by Osman et al. (2024) noticed significantly higher mortality rates in broilers supplemented with MOS-β-glucan at levels of 0.125 g/kg and 0.5 g/kg compared to the control group during the cumulative period. This suggests that certain specific doses of MOS might negatively affect poultry survival. Such discrepancies could be attributed to the differences in the experimental conditions, chicken strains, or environmental stress levels. Overall, the present findings suggest that prebiotics may exert a protective effect on broiler health.

For the feed conversion ratio (FCR), the data showed a significant improvement across all production phases, with a particularly notable enhancement observed during the growth phase. This improvement during that phase is critical, as it corresponds to a period of high nutritional demand and peak growth performance, which are essential for optimising poultry production profitability. These findings are in line with previous studies (Yang et al. 2007; Kamran et al. 2021; Fornazier et al. 2024) which demonstrated that the inclusion of MOSs significantly improved the FCR, especially during the growth phase. Furthermore, Osman et al. (2024) and Ding et al. (2019) reported significant improvements in FCR following MOS supplementation, supporting the current study’s results. Additionally, Yang et al. (2007) reported a 2% improvement in the FCR during the growth phase with MOS supplementation, while Tufail et al. (2019) observed a significant enhancement during the finishing phase with high-dose supplementation. Fornazier et al. (2024) reported a 7.3% improvement in the FCR at the optimal MOS dosage, and Polidoro et al. (2024) noted a 3% increase in the FCR performance compared to the control group. Similarly, Benites et al. (2008) reported a 1.99% improvement in FCR with MOSs, and Osman et al. (2024) found a significant increase in FCR during both the growth and finishing phases. Collectively, these results indicate that chickens receiving prebiotics were more efficient at converting feed into body mass, which is economically advantageous in poultry production.

The average weight gain was significantly improved across all production phases following the addition of MOSs in this study, with a particularly pronounced improvement during the growth phase. This improvement is particularly important, as this period marks a key stage when nutritional demands are high and rapid weight gain is crucial for meeting performance goals. These results are consistent with those of Tufail et al. (2019); Kamran et al. (2021); Fornazier et al. (2024); Polidoro et al. (2024). Tufail et al. (2019) reported that high-dose MOS supplementation significantly increased the weight gain at all phases. Benites et al. (2008) also showed a 2.79% improvement in body weight at 21 days and a 2.34% improvement at 42 days with MOS incorporation. Similarly, Park et al. (2019) observed a significant increase in body weight with a dose of 1 000 g/tonne of MOSs. Fornazier et al. (2024) reported a 5.36% increase in weight gain with the optimal addition of MOSs, while Polidoro et al. (2024) noted a 3.7% improvement in weight gain compared to the control group. Finally, Osman et al. (2024) also found a significant increase in weight gain with MOS supplementation at 1.0 g/kg. These results confirm the beneficial effect of MOSs, particularly during the growth and finishing phases, where nutritional needs are high.

The results of our study showed a significant increase in the number of lactic acid bacteria in chickens supplemented with the prebiotic MOS compared with the control group across all three intestinal segments (duodenum, ileum, and caecum) at all production stages. Our observations indicate that the prebiotic maintains its effectiveness throughout the entire intestinal tract. The significant increase observed in the duodenum is consistent with the work of Kim et al. (2011), who showed that supplementation with fructo-oligosaccharides (FOSs) at 0.25% and mannan-oligosaccharides (MOSs) at 0.05% significantly increased Lactobacilli populations in the small intestine. Furthermore, Jahanian and Ashnagar (2015) demonstrated that supplementation with MOSs at 0.1–0.2% also resulted in a significant increase in Lactobacilli numbers compared with the control group, thereby supporting our findings.

Regarding the caecum, our data also revealed a significant increase in the lactic acid bacteria, consistent with the work of Pourabedin et al. (2014), who reported a significant increase in this beneficial flora in the caecum of chickens supplemented with MOSs on days 16 and 26. Additionally, Abedin et al. (2014) observed that the MOS diet led to a significant increase in the populations of Lactobacilli and Bifidobacteria in the caecum. Furthermore, Baurhoo et al. (2009) noted that Lactobacilli concentrations in the caecum were significantly increased on days 24 and 34 following MOS supplementation, while Meesam et al. (2019) revealed a significant increase in the number of Lactobacilli in the caeca only on days 28 and 42, which is consistent with our results obtained on days 38 and 47. However, Biggs et al. (2007) found no notable effect of oligosaccharides on the Lactobacilli in the caecum at 21 days of age. Several factors can explain this lack of effect. On the one hand, the bacterial density in these segments is naturally higher, which may limit the additional impact of prebiotic supplementation. On the other hand, the longer digestive transit time in these parts of the intestine could reduce the bioavailability of the prebiotics, thus limiting their action on lactic acid bacteria.

In the ileum, although some authors, such as Kamran et al. (2021), did not observe significant differences, our study demonstrates a statistically significant improvement, suggesting that the effect of prebiotics may vary with dose, duration, and strain. In contrast, Wang et al. (2016) reported that by day 14, diets containing a prebiotic significantly improved the relative level of *Lactobacillus* in the ileal mucosa compared to negative control diets, highlighting that the effect of prebiotics can vary across intestinal segments and measurement periods. Furthermore, Osman et al. (2024) showed that groups receiving a diet enriched with MOS+β-glucan exhibited increased levels of yeast and *Lactobacillus*, while reducing *Enterococcus* levels. Finally, Teng and Kim (2018) reported that prebiotics promote the proliferation of beneficial bacteria such as Lactobacilli and Bifidobacteria, while reducing pathogenic bacteria, thereby reinforcing their role in enhancing gut health.

Although previous studies on prebiotic supplementation have yielded variable, sometimes contradictory results, our findings provide solid evidence that the specific combination of mannan-oligosaccharides (MOSs) and β-glucan (AGRIMOS^®^) can significantly improve key indicators in broiler chickens. Under our experimental conditions, prebiotic supplementation enhanced zootechnical performance by significantly improving body weight and feed conversion ratio, indicating improved feed efficiency. While the reduction in mortality was not statistically significant, a positive trend was observed in the treated group. Furthermore, enumeration of lactic acid bacteria revealed a significant enrichment in the prebiotic group across all the intestinal segments (duodenum, ileum, and caecum), suggesting the beneficial modulation of the gut microbiota. This finding contrasts with several previous studies that reported localised or inconsistent effects, highlighting the broader and more uniform modulation of the gut microbiota achieved in our trial. What distinguishes this study is its assessment of a commercial prebiotic product under conditions closely resembling those of the Algerian poultry industry, where the need for effective alternatives to antibiotics is both pressing and relevant. The observed benefits of AGRIMOS^®^ highlight its potential as part of a sustainable approach to poultry production, particularly in regions facing rising antimicrobial resistance. Further investigations are needed to examine the long-term effects and elucidate the mechanisms by which these compounds exert their influence across varying farming practices.
